# Gene Expression Correlates with Disability and Pain Intensity in Patients with Chronic Low Back Pain and Modic Changes in a Sex-Specific Manner

**DOI:** 10.3390/ijms26020800

**Published:** 2025-01-18

**Authors:** Maria Dehli Vigeland, Siri Tennebø Flåm, Magnus Dehli Vigeland, Manuela Zucknick, Monica Wigemyr, Lars Christian Haugli Bråten, Elisabeth Gjefsen, John-Anker Zwart, Kjersti Storheim, Linda Margareth Pedersen, Benedicte Alexandra Lie, the AIM Study Group

**Affiliations:** 1 Department of Research, Innovation and Education, Division of Clinical Neuroscience, Oslo University Hospital, 0450 Oslo, Norway; 2 Faculty of Medicine, University of Oslo, 0316 Oslo, Norway; 3 Department of Medical Genetics, Oslo University Hospital, 0450 Oslo, Norway; 4 Oslo Centre for Biostatistics and Epidemiology, University of Oslo, 0316 Oslo, Norway; 5 Department of Rehabilitation Science and Health Technology, Oslo Metropolitan University, 0130 Oslo, Norway

**Keywords:** chronic pain, low back pain, Modic changes, gene expression, RNA sequencing, sex differences

## Abstract

Chronic low back pain (cLBP) lacks clear physiological explanations, and the treatment options are of limited effect. We aimed to elucidate the underlying biology of cLBP in a subgroup of patients with Modic changes type I (suggestive of inflammatory vertebral bone marrow lesions) by correlating gene expression in blood with patient-reported outcomes on disability and pain intensity and explore sex differences. Patients were included from the placebo group of a clinical study on patients with cLBP and Modic changes. Blood was collected at the time of inclusion, after three months, and after one year, and gene expression was measured at all time points by high-throughput RNA sequencing. The patients reported disability using the Roland–Morris Disability Questionnaire, and pain intensity was assessed as a mean of three scores on a 0–10 numeric rating scale: current LBP, worst LBP within the last two weeks, and mean LBP within the last two weeks. The gene expression profiles were then correlated to the reported outcomes. Changes in gene expression over time correlated significantly with changes in both disability and pain. The findings showed distinct patterns in men and women, with negligible overlap in correlated genes between the sexes. The genes involved were enriched in immunological pathways, particularly T cell receptor complex and immune responses related to neutrophils. Several of the genes harbour polymorphisms that previously have been found to be associated with chronic pain. Taken together, our results indicate gender differences in the underlying biology of disability and pain intensity in patients with low back pain.

## 1. Introduction

Chronic low back pain (cLBP) is a common musculoskeletal disorder that affects people of all ages, genders and ethnicities, and a primary cause of disability and lost productivity worldwide [1]. It is estimated that one out of two adults will experience low back pain at some point in their lives, and more than 10% of patients with an acute episode will develop chronic pain (lasting for more than three months) [2]. For patients with cLBP, the underlying cause is often unknown, and the current treatment options have limited effect [3]. The patients are typically regarded within a biopsychosocial model recognizing that cLBP is a complex condition influenced by biological, psychological and social aspects, and their interaction with each other [4]. Advances in the research field depend on deeper knowledge of the biological component of cLBP, which is still poorly understood [5].

A subgroup of cLBP patients has Modic changes (MC), which are lesions in the lumbar intervertebral bone marrow identifiable by magnetic resonance imaging (MRI) [6]. Three MC types have been described, which seemingly represent interchangeable, but different stages of the pathological process [7,8]. MC type I corresponds to vertebral body oedema and likely an active inflammation, while MC type II is represented by fat infiltration in the bone marrow, and type III demonstrates sclerosis in the endplates [6].

Back pain as such has been estimated to have a heritability of 30–68% [9,10,11]. Two large genome-wide association studies (GWAS) have been performed, but only four genetic variants have been reliably associated with back pain [12,13]. Interestingly, sex-specific genetic associations have later been observed in one of these datasets [14], which revealed two and seven loci associated with back pain in males and females, respectively. Even more GWAS have been performed in the wider phenotype, chronic pain [15,16,17,18], also here with clear sex differences [19]. Most variants identified are common variants situated in non-coding regions in the genome, potentially affecting gene expression.

In the last decades, an increasing body of research has demonstrated how pain differs in men and women [20]. A large part of chronic pain conditions, including musculoskeletal pain, are more prevalent in females, who typically also report greater pain sensitivity and higher pain scores than men [21,22]. The sexes, furthermore, respond differently to certain treatments [20]. Several causal factors are indicated, with physiological aspects, sex hormone levels and neuroimmunological distinctions as key concepts [23]. Correspondingly, research suggests that there are significant differences in cLBP prevalence, severity, and comorbidities between men and women, with women reporting worse rates in all the mentioned aspects [24,25]. Despite this, sex differences are not typically considered in cLBP research, diagnosis, or treatment [26].

In this study, we investigated whether gene expression patterns were associated with pain intensity and disability in peripheral blood from a phenotypically homogeneous group of cLBP patients having the inflammatory MC type I, and explored potential sex-differences.

## 2. Results

### 2.1. Study Cohort Characteristics

All eligible patients with MC type I and blood sampling at three time points receiving placebo treatment in the AIM study were included in the analyses (*n* = 41, Supplementary Table S1A). No significant differences were observed in the clinical characteristics between female and male patients at the time of inclusion, except for disease duration and LBP intensity (Table 1).

**Figure 1:**
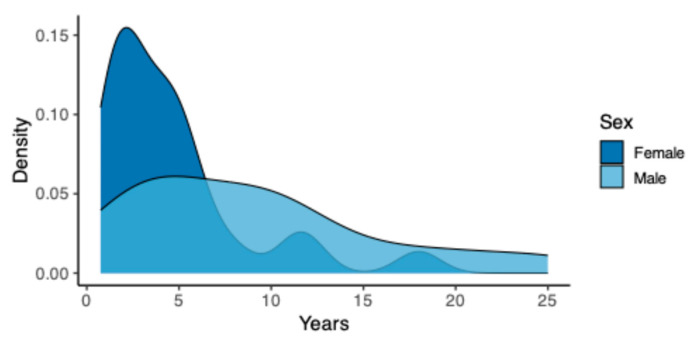
Distribution of duration of symptoms in years, separated by sex.

The female patients had a shorter LBP duration (mean 4.6 years) than men (mean 9.1 years) (Figure 1) and slightly less pain intensity and disability (Supplementary Figure S1). There was little improvement over time observed in either sex (Supplementary Figure S1).

### 2.2. Strong Correlation Between RMDQ and NRS Scores

**Figure 2:**
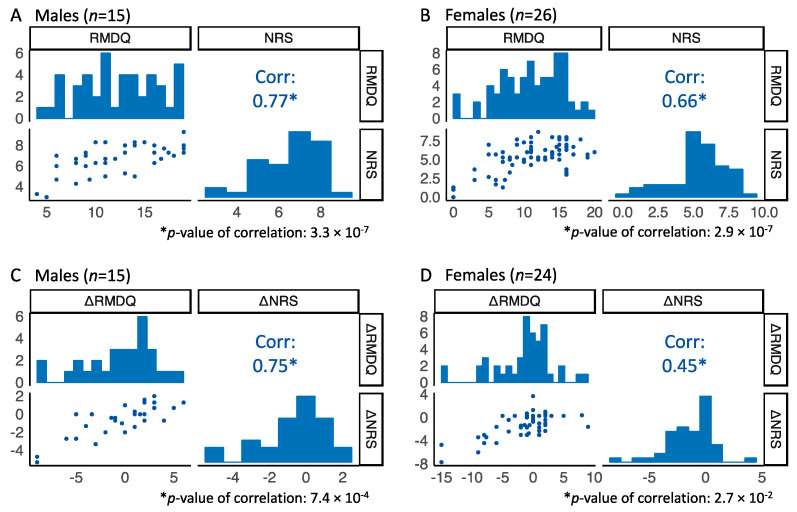
Marginal distributions and repeated measures correlation between (**A**,**B**) RMDQ and NRS from all three time points and (**C**,**D**) between ΔRMDQ and ΔNRS. The latter plots include values from two time intervals, day 0–day 100 and day 0–1 year.

The Roland–Morris Disability Questionnaire (RMDQ) scores and the numerical rating scale (NRS) pain intensity scores generally correlated strongly in the study population (Figure 2A,B). The correlations were slightly stronger in males than females, with repeated measures correlation coefficients of 0.77 and 0.66, respectively. The observed skewness in the NRS distribution, especially pronounced in females (Figure 2B), is explained by the inclusion criteria of NRS > 5 in the clinical study.

A similar pattern was observed when testing the delta (Δ) values between time points (Figure 2C,D). The correlation between ΔRMDQ and ΔNRS was stronger in males, with a repeated measures correlation coefficient of 0.75 compared to 0.45 in females.

### 2.3. Gene Expression Levels Associated Poorly to Disability or Pain at Single Time Points

Assuming that the samples from different time points represented replicate measurements in the individual patients, we tested whether the NRS or RMDQ values were associated with gene expression levels at single time points across individuals. No significant associations (False discovery rate (FDR) < 0.05) were observed in the total cohort to neither RMDQ nor NRS, with or without controlling for sex. In the sex-stratified analyses, three associations were revealed (Supplementary Table S2). The only two significant findings in males were *RNY3P1* (padj = 0.02) and *METTL24* (padj = 0.01), whose increased expression associated with respectively decreasing and increasing scores of RMDQ. In females, a higher expression of *TNKS2-AS1* solely associated with decreasing scores of NRS. Taken together, few associations between gene expression levels and pain intensity or disability at single time points were seen, which could be due to inter-individual differences in the use of the pain scores.

### 2.4. Sex-Specific Associations Between Changes in Gene Expression and ΔRMDQ and ΔNRS

As the intra-individual reproducibility and reliability of pain scores are high [27,28], the score is most valuable when addressing individual change in pain. We, therefore, investigated how change in RMDQ and NRS scores, i.e., ΔRMDQ and ΔNRS, were associated with change in gene expression, ΔGEX, within individuals. First, we tested for association in the total population, adjusting for sex in the model. No significant association to either ΔRMDQ nor ΔNRS were observed, except one uncategorised gene *ENSG00000279631* to ΔNRS (padj = 0.002).

Given known biological differences in pain between sexes, we next included sex as an interaction term in the model, which provided statistical evidence for significant differences between the sexes (Supplementary Table S3). This difference was observed for ΔNRS, but not ΔRMDQ. The genes showing significantly more positive correlation in males than females were associated with GO terms T cell receptor complex (adjusted *p*-value (padj) = 4 × 10^−19^) and adaptive immune response (padj = 6 × 10^−6^) (Supplementary Table S10), and included T cell genes like *CTLA4*, *CD28*, *IL23A*, *CD3D* and *TRAV4*, (Supplementary Figures S2 and S4B). In contrast, genes showing more positive correlation in females than males were associated with inflammatory response (padj = 4 × 10^−4^), response to cytokine (padj = 4 × 10^−3^), and protein kinase activity (padj = 6 × 10^−3^), and most genes showed expression exclusively (e.g., *IL1R2*, *MME*, *CXCL8* and *MMP9*), or predominantly (e.g., *STAT3*) (Supplementary Figure S3) in neutrophils according to the Human Protein Atlas.

We followingly performed the association analysis in sex-stratified cohorts. With an FDR cut-off at 0.05, 90 genes associated significantly with ΔRMDQ in males and 3 in females (Supplementary Tables S4 and S5). More genes had expression changes that were associated with ΔNRS, i.e., 864 genes in males and 63 in females (Supplementary Tables S6 and S7). Among the significant findings were *METTL24* and *TNKS2-AS1*, genes we already identified to associate with RMDQ and NRS scores at single time points in, respectively, males and females.

**Figure 3:**
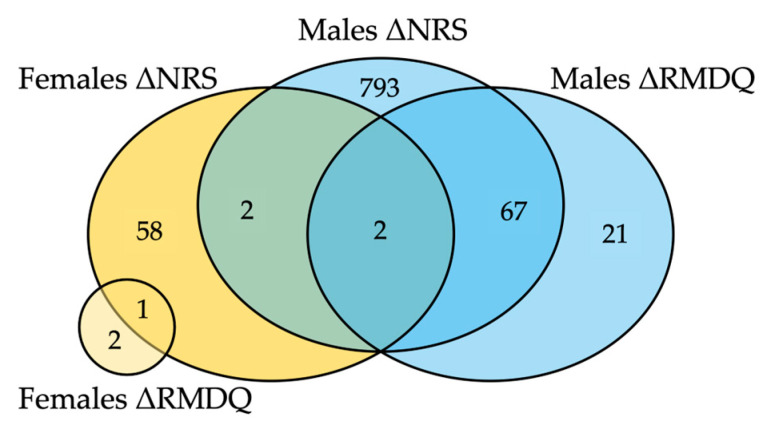
Diagram showing the number of genes with significant association between change in gene expression (ΔGEX) and change in disability/pain intensity (ΔRMDQ/ΔNRS) in males and females (FDR < 0.05).

Several genes were associated with both ΔRMDQ and ΔNRS (Figure 3). Only four genes overlapped between the sexes, with two genes associating with ΔNRS in both females and males and two also with ΔRMDQ in males (Figure 3 and Supplementary Figure S4). Interestingly, three of these (*TRAV4*, *MRPL32* and *PIK3R5*) demonstrated associations of opposite directions in the sexes (Supplementary Figure S4B–D). Hence, only one transcript showed an association in the same direction in both females and males, i.e., the long non-coding RNA *LLNLR-246C6.1* (Supplementary Figure S4A). Genes whose expression significantly increased with increasing pain intensity in males were again associated with the GO term T cell receptor complex (padj = 7 × 10^−4^), which also was significant for increasing disability in males (padj = 1 × 10^−2^). The significant genes from the female analyses were not enriched for any relevant GO-terms.

**Figure 4:**
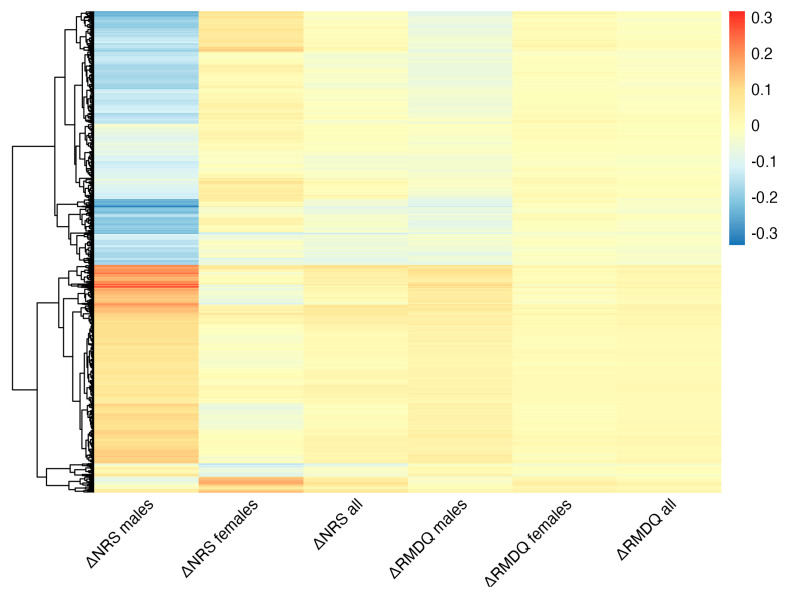
Heatmap showing genes with significant (padj < 0.1) association between ΔGEX and either ΔRMDQ or ΔNRS in at least one analysis (946 genes). Each row corresponds to a gene, and the colours represent the correlation coefficient. The figure demonstrates clear sex-specific patterns, especially evident in the first two columns.

Sex-specific patterns were observed through heatmap clustering of all genes with significant association between ΔGEX and either ΔRMDQ or ΔNRS (padj < 0.1) in at least one cohort (Figure 4). The figure demonstrates clear sex-specific patterns, especially evident for ΔNRS in males versus females (the first two columns of Figure 4). The overall stronger correlations observed to ΔNRS than ΔRMDQ reflect the higher number of significant associations to this score.

### 2.5. Genes with Risk Variants for Chronic Pain Associated with ΔNRS

**Figure 5:**
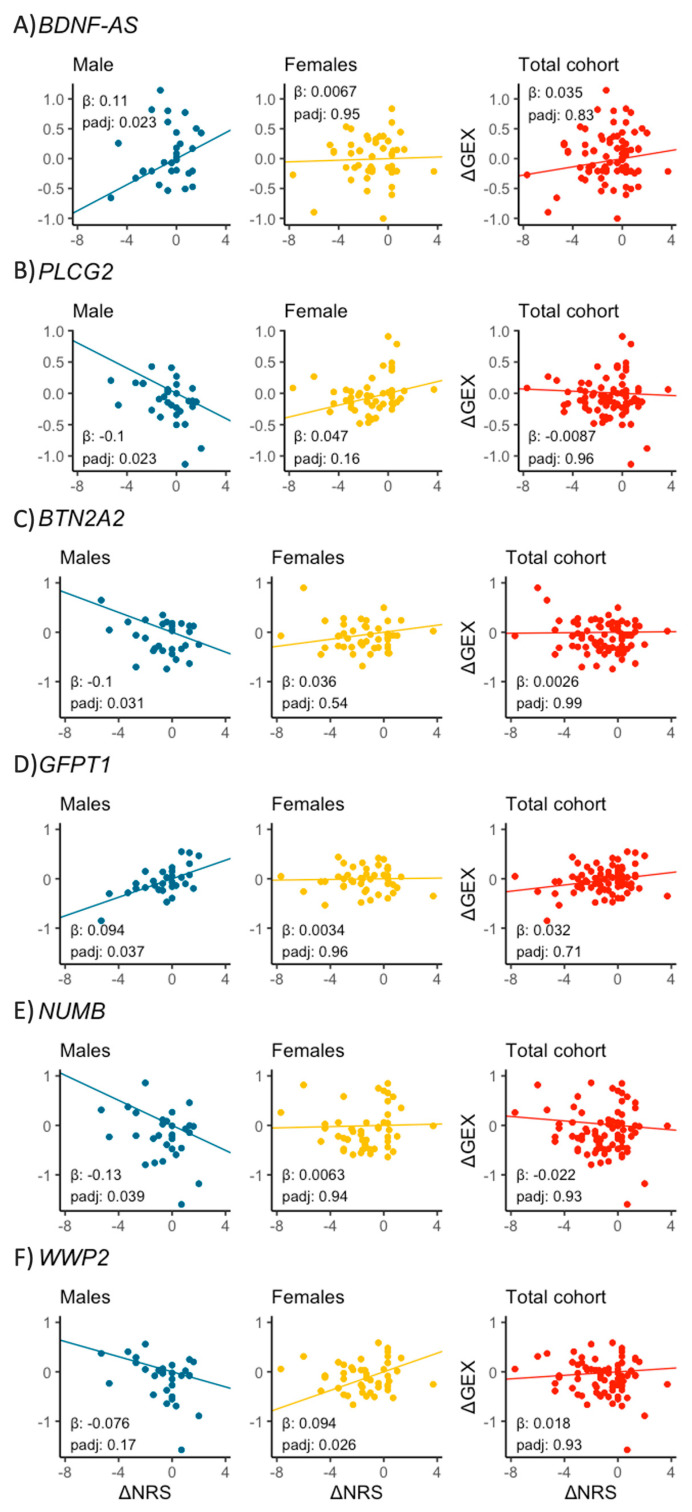
Correlation between ΔGEX and ΔNRS in males, females, and the total mixed-sex cohort for genes reported in GWAS studies to be associated with chronic pain: (**A**) *BDNF-AS*; (**B**) *PLCG2*; (**C**) *BTN2A2*; (**D**) *GFPT1*; (**E**) *NUMB* and (**F**) *WWP2*.

Among the genes with changes in expression that were associated with changes in pain score (FDR < 0.05) exclusively in males or females, six were previously reported to harbour polymorphisms associated with chronic pain through GWAS: *BDNF-AS*, *GFPT1, PLCG2*, *BTN2A2*, *NUMB* in males, and *WWP2* in females (Figure 5 and Supplementary Table S9). Neither of the genes had been reported as sex-dependent GWAS associations. However, two of the genes with significantly different correlation of ΔGEX and ΔNRS between the sexes (identified in our analysis applying sex as an interaction term), *NPM1* and *FALEC*, were previously reported as sex-dependent GWAS associations to chronic pain in females (Supplementary Figure S5).

When relaxing the significance level (FDR < 0.1), we found that expression of several additional GWAS genes correlated with pain intensity (i.e., 14 in males and 6 in females) and disability (i.e., 5 in males) (Supplementary Table S9). Among these was the mentioned *NPM1* observed in both males and females, with correlations to ΔNRS of opposite sign in the sexes (Supplementary Figure S5A). Expression of the gene *SCIN*, harbouring polymorphisms previously reported as a suggestive association to LBP with MC, correlated negatively with ΔNRS in females exclusively.

Investigating candidate genes known to be relevant for pain conditions, we found that expression of interleukin 6 (*IL-6*) had levels positively correlating with ΔNRS in males (Supplementary Figure S6). We did not observe significant correlations with other established pain genes, including the much-studied *COMT* gene (Supplementary Figure S7).

## 3. Discussion

To our knowledge, this is the first study investigating how patient-reported disability and pain intensity over time correlates with gene expression levels in blood. The observed significant correlations were highly specific for males or females in our patient population with cLBP and MC type I.

Importantly, RMDQ/NRS scores at single time points showed few associations to the gene expression levels. This likely reflects the subjective nature of the RMDQ/NRS measures, as it has been demonstrated that individuals exposed to stimuli of the same intensity report diverse pain levels [29,30]. Inter-individual differences in pain can partly be predicted by objective measures like brain morphology and genetics, suggesting true biological differences in the pain experience of an individual [29,31,32]. Even if individual reference scales for pain might vary, repeated assessment have shown high intra-individual reliability [27,33].

Indeed, our results showed that change in expression levels correlated significantly with both changes in disability and pain intensity for several genes, although in a clear sex-dependent manner. No correlations were observed in the total cohort, while there were multiple significant findings in sex-segregated analyses. Furthermore, several genes showed correlations of opposite direction in the sexes. Sex-differences in pain are documented both clinically and experimentally [34,35,36]. Chronic pain diagnoses have a higher prevalence in women [21], and women report enhanced sensitivity and greater pain in most experimental pain models [37]. A range of causes have been proposed, including effects of sex hormones [38,39], genetics [40,41], and differences in psychosocial factors like pain-coping strategies [42] and stress at young age [43]. As it is possible that the observed sex differences could partly be related to sex hormone levels [20,39], future studies should include measurements of hormone levels to further examine this possibility. Of note, this study only considered biological sex. We detected considerably more gene expression correlations with pain and disability in males than females. The reason for this is unclear. As we had markedly less males than females in our study cohort (15 vs. 26), this is unlikely a consequence of imbalance in statistical power. A possibility is that pain mechanisms in males involve biological factors or pathways that are more easily detectable in blood. Our male cohort also had longer disease duration. Pain with a long duration might have a stronger biological signature, particularly in peripheral blood, and hence, the effects in females had perhaps not yet manifested. Such a relationship between disease duration and biology is yet to be described; however, comparing acute and chronic pain, more genetic associations are found to chronic pain [44].

The samples in this study were included from the placebo group of a clinical trial, and we cannot rule out a possible impact of the placebo treatment in our analysis. Research has shown that sex differences exist in response to placebo treatment, with males generally exhibiting a stronger placebo response compared to females [45]. However, in our study, we did not observe such a pattern. Males rather tended to improve slightly less than females in terms of both pain and disability. As the strongest correlations in our results were observed in males, we, therefore, do not believe that the observed sex differences can be attributed to variations in effect of the placebo treatment. However, pain amelioration and placebo response are biologically intertwined and could share biological mechanisms including those observed in our study.

We uncovered associations between change in gene expression and pain intensity in several genes previously associated with chronic pain in GWAS. *BDNF-AS* [18], *GFPT1* [46] and *WWP2* [47] correlated positively, while *PLCG2* [48], *BTN2A2* [49] and *NUMB* [15,19] correlated negatively. The correlations were strictly sex-specific, and all but one (*WWP2*) were only observed in men. A less stringent significance cut-off (FDR < 0.1) revealed several additional correlations, and the only gene (*NPM1* [19]) observed in both males and females demonstrated correlations of opposite directions. Furthermore, the expression of *NPM1* and *FALTEC*, genes previously reported as sex-dependent GWAS associations to chronic pain [19], had significantly different correlation to changes in pain intensity between the sexes in our study. This suggests that current studies on the genetics and biology of chronic pain could lose statistical power and draw incomplete conclusions if sex is not included as a covariate.

We next observed significant positive correlations between the expression of *IL-6* and pain intensity in males. The same pattern was observed for disability, although not significant. This is noteworthy, as IL-6 is an inflammatory cytokine involved in a variety of immunological conditions and pain mechanisms [50]. Increased serum levels of IL-6 are observed in patients with LBP [51] and are associated with a chronic outcome [52]. On the other hand, our study did not show any significant findings involving *COMT*, which has been widely associated to both clinical and experimental pain responses [53,54].

Most genes (66%) with expression levels associated with disability in males, were also associated with pain intensity. This was not surprising, as the two scores were strongly correlated, especially in males. Little overlap of the gene expression associations was seen in females probably due to weaker correlation between pain and disability and much fewer significant findings. A substantial overlap between NRS and RMDQ findings was expected, as RMDQ mostly reflects the physical aspects of LBP-related disability [55]. Interestingly, we found more gene expression associated with pain intensity than disability, which could indicate that pain better represents the biological component of cLBP, at least with MC type I. The lack of associations with ΔRMDQ could also reflect the stronger association between disability and MC type II [56], whereas MC type I has clinically been linked to inflammatory pain like morning stiffness [57].

We observed two distinct biological pathways in the genes differentially correlated in males vs. females. One involving T cells and the other involving inflammatory responses via neutrophils. Intriguingly, distinct immune cells have been demonstrated to be involved in the mechanisms of pain in the sexes [58], and several immune cells have been implicated. Microglia and T cells have been shown to promote pain hypersensitivity in a testosterone-dependent manner in, respectively, male and female rats [59]. Immune cells likely contribute to the transition from acute to chronic pain, e.g., infiltrating neutrophils, macrophages and T cells communicate with resident astrocytes, microglia and oligodendrocytes in the central nervous system to release mediators that sensitize nociceptors [58,60]. Whether our results are a consequence of distinct immune cell involvement or divergent molecular pathways is not clear, but variations in blood cell type proportions in the samples from each patient have previously been shown to be negligible [61].

Taken together, our results indicate sex differences in biological mechanisms related to pain, thus confirming the importance of including sex as a covariate in pain research. Specifically, changes in pain intensity and disability significantly correlated with changes in gene expression in blood from patients with cLBP and inflammatory MC type I, implicating distinct immunological pathways in the pain experienced by men and women.

## 4. Materials and Methods

### 4.1. Study Cohort

This study included patients enrolled in a randomised, placebo-controlled trial assessing the efficacy of 100 days of amoxicillin treatment in patients with cLBP and MC (the AIM study) [62]. Only the patients receiving placebo treatment were included in the current study, approximating an untreated patient population. Additionally, only patients with the most inflammatory MC type (MC type I), of Caucasian ethnicity, and with successful blood sampling were included. The patients suffered from substantial LBP at the time of inclusion (intensity ≥ 5 on a NRS from 0 (no pain) to 10 (worst pain imaginable) and had little other comorbidity. Eligibility criteria and the study protocol for the AIM study are published elsewhere [63]. Written informed consent was obtained from all patients in accordance with the Helsinki Declaration, and the study was approved by the Regional Ethics Committee in South-East Norway (project 2014/158). A patient representative was included in the AIM study scientific board and was involved in all major decisions.

### 4.2. Study Design

Patient-reported outcome measures and gene expression from blood collected at the following three time points were included in this study: (1) before start of intervention (day 0), (2) at the end of intervention (day 100), and (3) at 1-year follow-up (1 year). As these patients only received a placebo treatment, we considered the intervention to have a negligible effect on the blood samples. We consider it unlikely that the patients were at parallel disease stages, considering how LBP symptoms show a range of different development patterns over time [64,65] and the great variation in duration of symptoms between the patients. The time independence of our samples was confirmed through preliminary analysis, which showed that the time variable had no significant effect on the gene expression data. This is in line with previous analysis on these patients [61]. Consequently, we regarded the samples from the three time points as biological replicates effectively independent of time.

### 4.3. Patient Characteristics

Patient background characteristics were collected at day 0, including information on age, sex, BMI, ethnicity, smoking habits, information regarding LBP duration, and different blood parameters.

### 4.4. Patient Reported Outcomes on Disability and Pain Intensity

The patients’ level of disability and pain intensity was assessed using two patient-reported outcome measures: the Norwegian validated version of the RMDQ [55], with 24 questions evaluating the patients LBP-related disability during everyday activities, and the mean of three NRSs measuring the intensity of their LBP (current LBP, worst LBP within the last two weeks, and mean LBP within the last two weeks).

Data on one or two out of the 24 questions in the RMDQ questionnaire were missing at one time point for seven patients. The missing values were imputed by taking the mean of the other questions from the same patient. After imputation, only one female patient lacked a total RMDQ score, as well as NRS on day 100 and 1 year. A second female patient lacked an NRS score from day 0 (Supplementary Table S1A).

The correlation between the RMDQ and NRS values was evaluated with repeated measures correlation using the rmcorr v.0.7.0 R package [66].

### 4.5. Blood Sampling and Preparation of RNA

Peripheral blood for gene expression was sampled in Tempus Blood RNA Tubes (Thermo Fisher Scientific, Waltham, MA, USA). Total RNA was isolated from the blood using the Preserved Blood RNA Purification Kit I (Norgen Biotek, Thorold, ON, Canada) according to the manufacturer’s instructions. DNAse treatment was included as recommended. RNA quality and concentration were measured using the BioAnalyzer 6000 Nano kit (Agilent Technologies, Santa Clara, CA, USA) and Qubit RNA HS (Thermo Fisher Scientific), to a mean concentration of 151 ng/μL and mean RNA integrity number (RIN) of 9.1. The total RNA samples were depleted for ribosomal RNA and globin transcripts with the Globin-Zero^®^ Gold rRNA Removal Kit or the TruSeq Stranded Total RNA with Ribo-Zero Globin kit (Illumina, San Diego, CA, USA).

### 4.6. Generation of Gene Expression Data

RNA was prepared for sequencing using TruSeq RNA library prep kits (Illumina) and sequenced with 2 × 75 bp paired-end configuration on the HiSeq3000 platform (Illumina) to a mean of 62 million reads per sample. The quality of the raw sequencing reads was assessed using FastQC and Qualimap [67,68]. The reads were mapped to the human genome (GRCh38.p10) using HISAT2 v2.1.0 [69] and counted with featureCounts from Subread v1.6.3 [70], using gene coordinates from Ensembl 88 [71]. Of the sequencing reads, 97.3% were successfully aligned to the reference genome, and 46.6% of these were assigned to genes. Only autosomal genes were kept for the analyses, and lowly expressed genes were filtered out using the R package edgeR v4.2.0 [72]. Data from 21 837 genes remained for downstream analysis, with a mean library size of 35 million (range 11–93 million). The raw counts were normalised using edgeR’s trimmed mean of M-values method. The generation and quality control of the sequencing data are described in further detail elsewhere [73].

### 4.7. Statistical Analyses

#### 4.7.1. Differential Gene Expression Analysis

We analysed the correlation between gene expression levels and the RMDQ and NRS scores using the R package limma v3.54.1 [74,75], controlling for sex, age, duration of LBP symptoms and sequencing batch effects. The intra-individual variation in the repeated measurements of the patients were included in the model as a random effect using the duplicateCorrelation function. As limma assumes normally distributed data, the read counts were log2-transformed prior to the analysis, using the integrated voom function [76]. *p*-value distributions were estimated empirically from the t-scores using the Bayesian method implemented in the bacon v1.26.0 R package [77] to correct for inflation in the test statistics, before controlling for the FDR at 0.05 using the Benjamini–Hochberg method [78].

In order to scrutinize the effect of sex, we performed the DGE analysis both in the total study population, with and without controlling for sex, and in the sex-stratified cohorts separately.

#### 4.7.2. Association of Change in Gene Expression and Disability and Pain Intensity

In addition to testing the associations between RMDQ/NRS and gene expression at single time points, we next tested whether changes in gene expression (ΔGEX) correlated with changes in RMDQ and NRS (ΔRMDQ and ΔNRS) over time. Two time intervals were considered: (1) day 0 to day 100 and (2) day 0 to 1 year. Change in gene expression was calculated from gene expression counts normalised for library size and RNA composition effect using the varianceStabilizingTransformation function implemented in the R package DEseq2 v1.38.3 [79].

Associations between ΔGEX and ΔRMDQ/ΔNRS were computed using linear mixed models with the lmer function in the R package lme4 [80], according to the following model (using the notation used in R): Δ*GEX* ~ 1 *+* Δ*RMDQ* + *age* + *sex* + *batch* + *sympt. duration* + (1|*ID*), where *batch* represents the sequencing batch effect, and *sympt. duration* the LBP duration in years. ΔNRS was analysed likewise. The FDR was controlled at 0.05 using the Benjamini–Hochberg method. As in the DE analysis described above, various treatments of the sex variable were used, both testing for significant differences between the sexes in the total study population and with separate analysis of the male and female cohorts. General correlation patterns were explored through correlation heatmaps with complete linkage hierarchical clustering using the R package pheatmap v1.0.12.

We next investigated whether our significant findings had been previously linked to chronic pain or back pain through investigation of GWAS-reported genetic associations, additionally including an in-house list of other plausibly relevant genes (see Supplementary Table S8 for a complete list).

The significant correlations were investigated for functional enrichment using g:Profiler (version e111_eg58_p18_f463989d) with g:SCS multiple testing correction method applying significance threshold of 0.05 [81]. The cell-specific expression of the genes was explored using the Human Protein Atlas, proteinatlas.org [82].

## Figures and Tables

**Table 1:** Demographic and clinical characteristics of patients (n = 41) at day 0.

	Female (*n* = 26)	Male (*n* = 15)	Pnc
Age (mean, SD)	47.4 (8.9)	45.1 (9.3)	0.45
BMI (median, IQR)	24.7 (3.0)	26.7 (4.5)	0.15
Smoking, *n* = 39	15%	13%	0.89
Previously operated for disc herniation	23%	13%	0.44
LBP duration in years (mean, SD), *n* = 40	4.6 (4.0)	9.1 (6.9)	0.03 *
Glucose, mmol/L (mean, SD)	5.1 (0.7)	5.1 (0.5)	0.62
Thrombocytes, ×10^9^/L (mean, SD)	268.1 (73.5)	232.5 (79.5)	0.17
Haemoglobin, g/100 mL (mean, SD)	13.5 (1.0)	15.2 (0.9)	NA
Haematocrit, % (mean, SD)	0.4 (0.0)	0.4 (0.0)	NA
Creatinine, µmol/L (mean, SD)	63.7 (9.9)	84.3 (13.1)	NA
AST, U/L (mean, SD), *n* = 40	21.2 (4.0)	26.0 (6.1)	NA
WBC, ×10^9^/L (mean, SD)	6.8 (1.4)	6.0 (2.4)	0.29
Disability, RMDQ (mean, SD)	12.1 (3.5)	12.7 (4.2)	0.67
LBP intensity, NRS (mean, SD), *n* = 40	6.3 (1.2)	7.1 (0.9)	0.03 *
CRP, U/L (mean, SD)	1.7 (2.3)	1.5 (1.4)	0.66
Neutrophils (%, SD), *n* = 40	59.0 (8.8)	58.5 (10.9)	0.88
Lymphocytes (%, SD), *n* = 40	31.7 (8.2)	30.4 (9.8)	0.69
Monocytes (%, SD), *n* = 40	6.9 (2.2)	8.1 (2.3)	0.11
Eosinophils (%, SD), *n* = 40	1.9 (1.0)	2.3 (1.8)	0.36
Basophils (%, SD), *n* = 40	0.6 (0.6)	0.7 (0.5)	0.78

AST: Aspartate aminotransferase; BMI: Body mass index; CRP: C-reactive protein; IQR: Interquartile Range; LBP: Low back pain; NRS: Numerical rating scale; RMDQ: Roland–Morris Disability Questionnaire; SD: Standard deviation; WBC: White blood cell count. Pnc: Uncorrected *p*-values. NA: Not applicable, clinical values with expected different levels in males and females. *: Significant with *p* < 0.05.

## Data Availability

Raw gene expression count matrices are available as GEO accession GSE285917.

## Supplementary Materials

The following supporting information can be downloaded at: https://www.mdpi.com/article/10.3390/ijms26020800/s1.
