# Supplementary material for: Gene Expression Correlates with Disability and Pain Intensity in Patients with Chronic Low Back Pain and Modic Changes in a Sex-Specific Manner
Source: Int J Mol Sci. 2025 Jan 18;26(2):800. doi: 10.3390/ijms26020800 (PMC11766089; doi:10.3390/ijms26020800)
Supplement: Supplementary file 1 [file ijms-26-00800-s001.zip › Supplementary Figures.pdf]

Supplementary Figures S1–S7

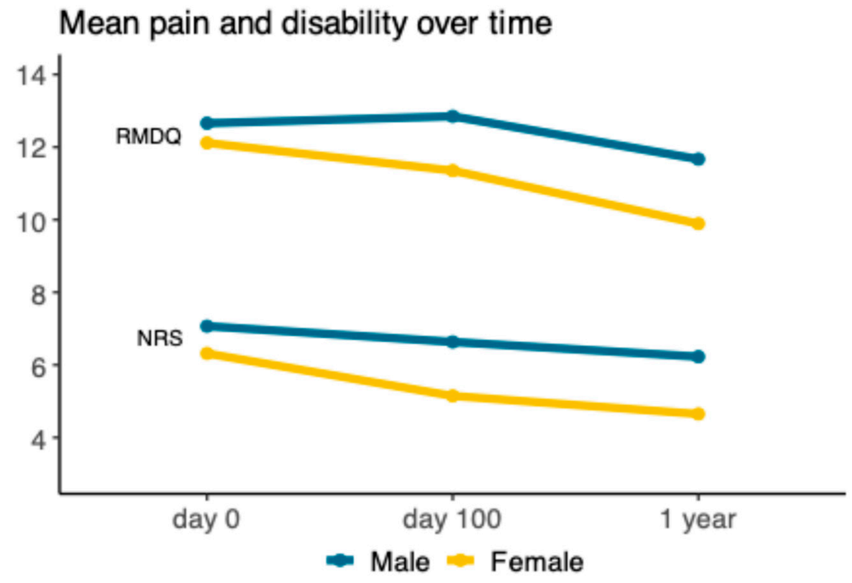

Supplementary Figure S1: Mean RMDQ and NRS over time in males and females.

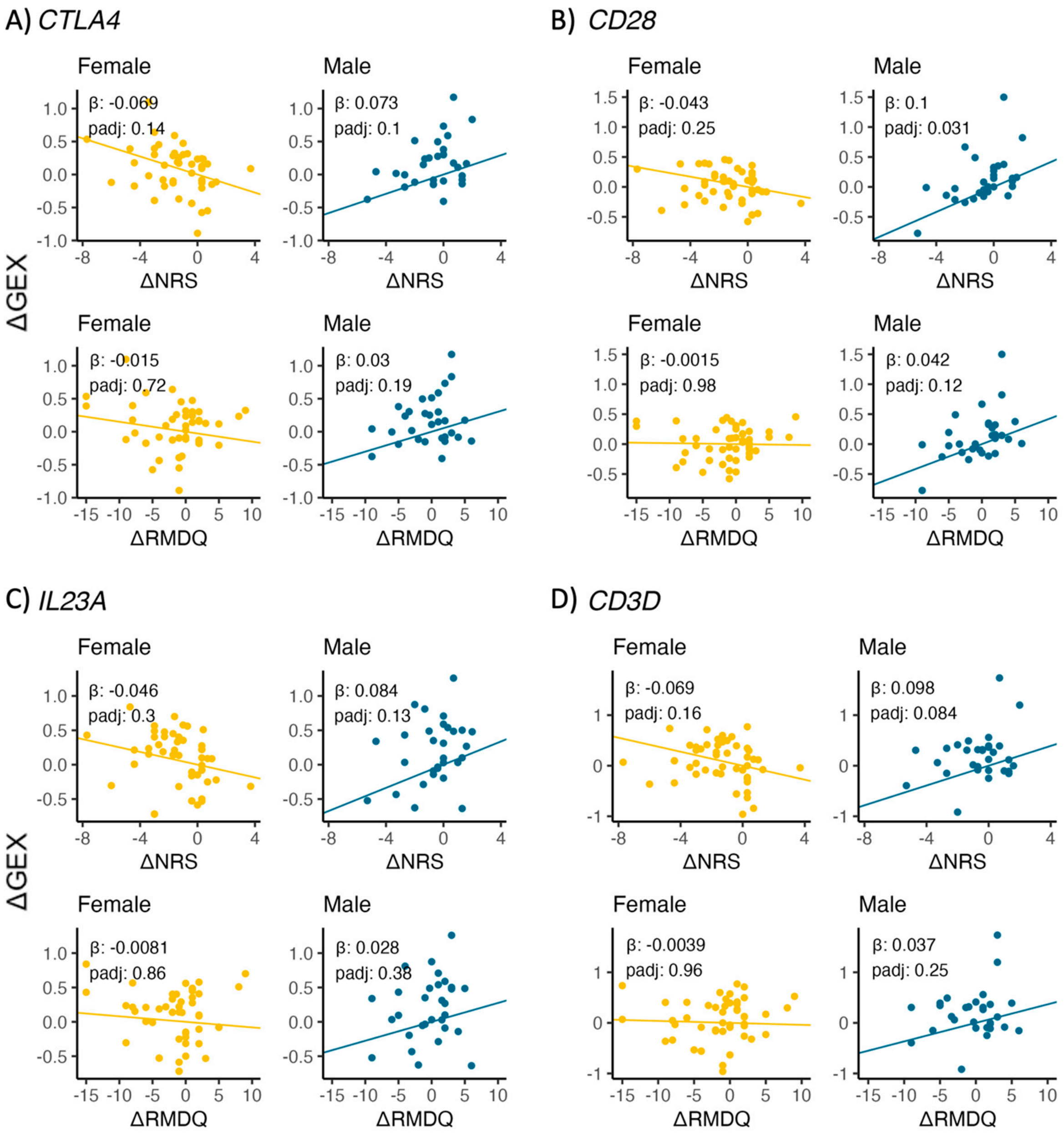

Supplementary Figure S2: A selection of the genes contributing to the enrichment of GO terms «T cell receptor complex» and «adaptive immune response» showing significantly more positive correlation with ΔNRS in males than females.

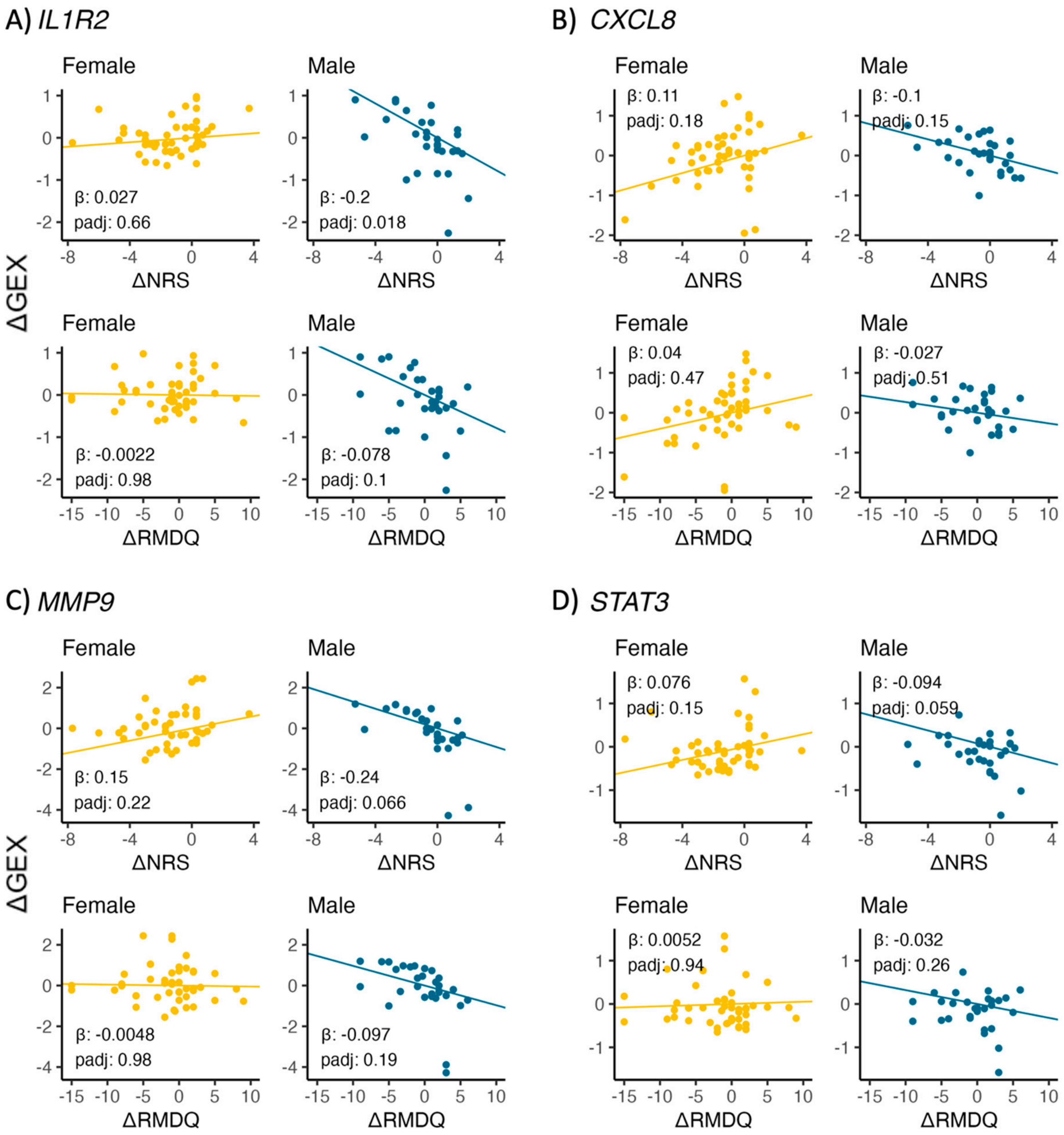

Supplementary Figure S3: A selection of the genes contributing to the enrichment of the GO term «inflammatory response» (p=4x10^{-4}) showing significantly more positive correlation with ΔNRS in females than males.

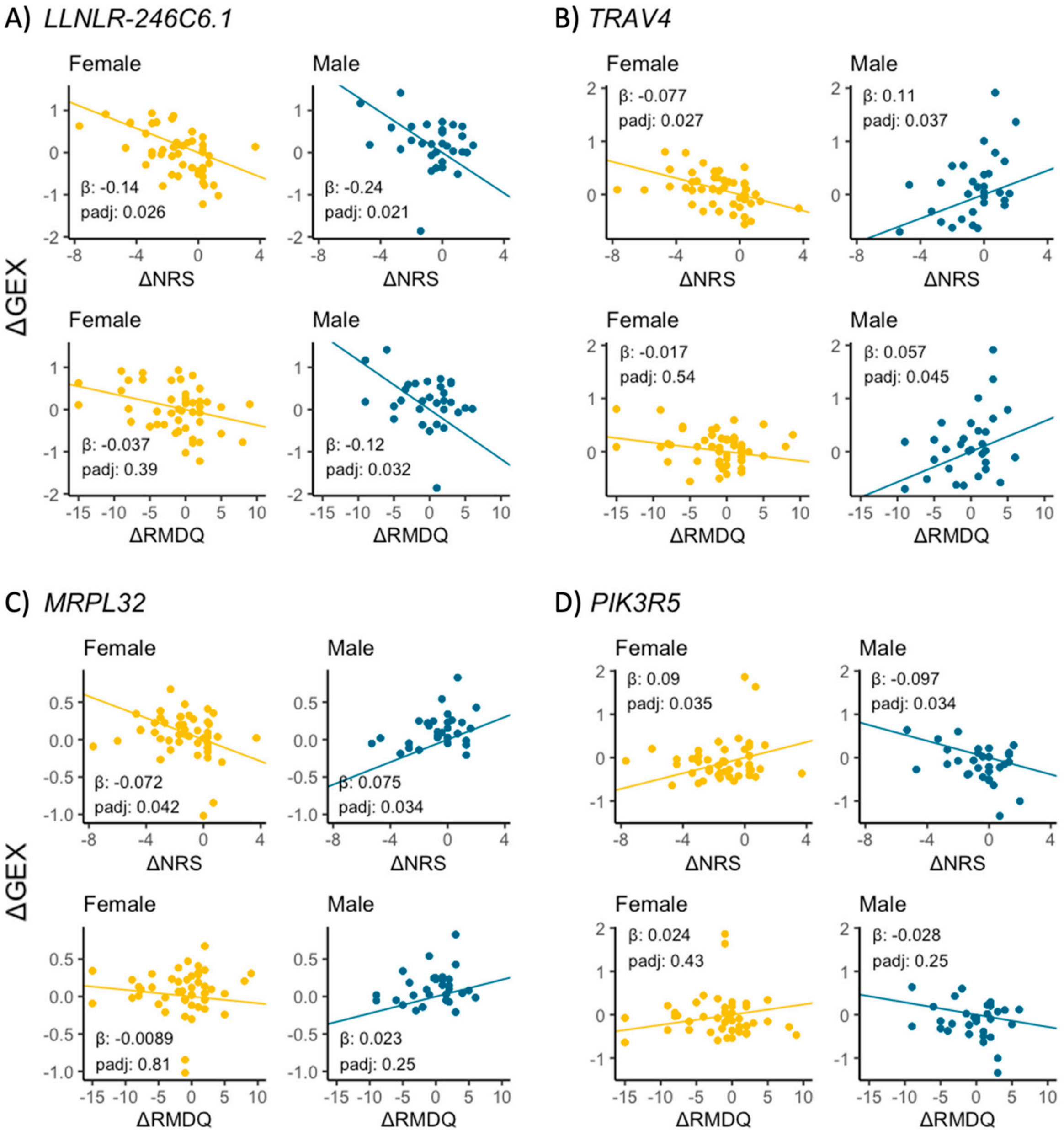

Supplementary Figure S4: Correlation between ΔNRS and ΔRMDQ and change in expression for LLNLR-246C6.1 (A), TRAV4 (B), MRPL32 (C) and PIK3R5 (D) in females and males.

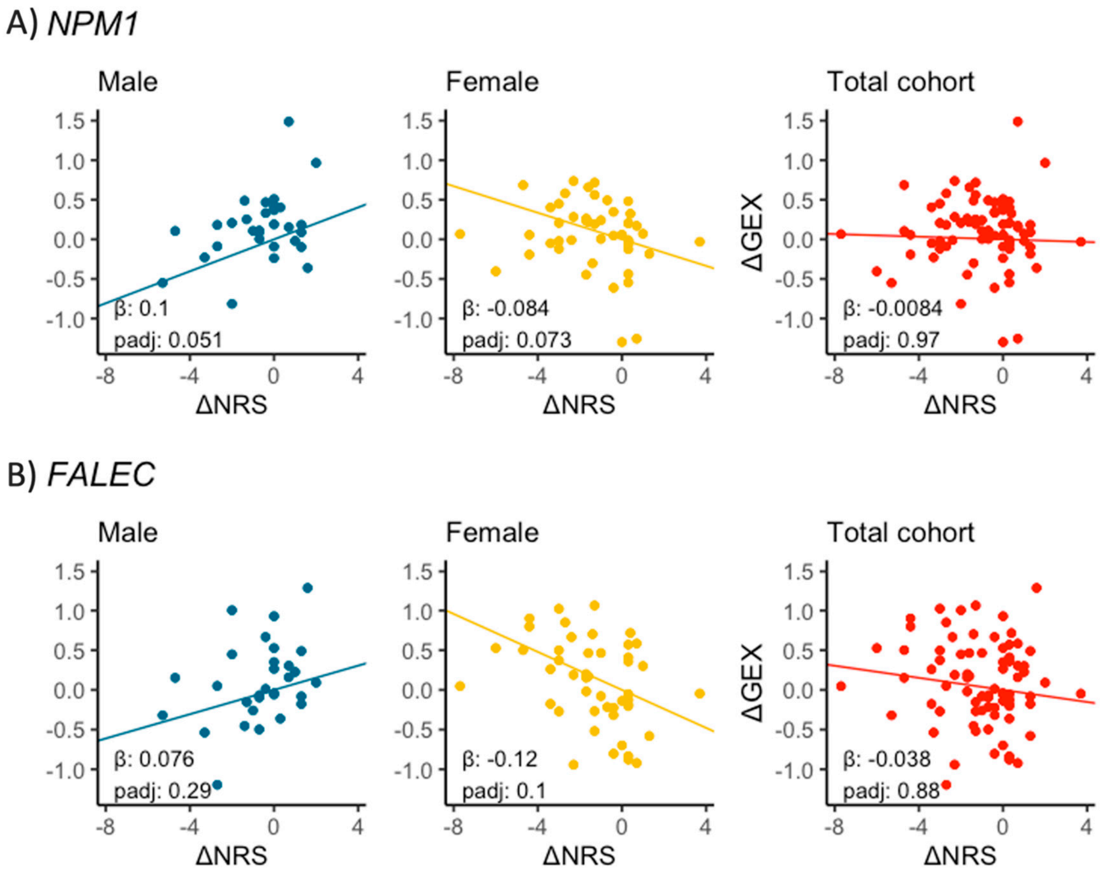

Supplementary Figure S5: Correlation between ΔNRS change in expression for NPM1 (A) and FALEC (B) in males, females, and mixed-sex cohorts. NPM1 show correlations to ΔNRS in both sexes with significant difference between the sexes. Also FALEC demonstrated significant difference between the sexes, but significant correlations were not directly observed in the sex-segretated analysis.

IL-6

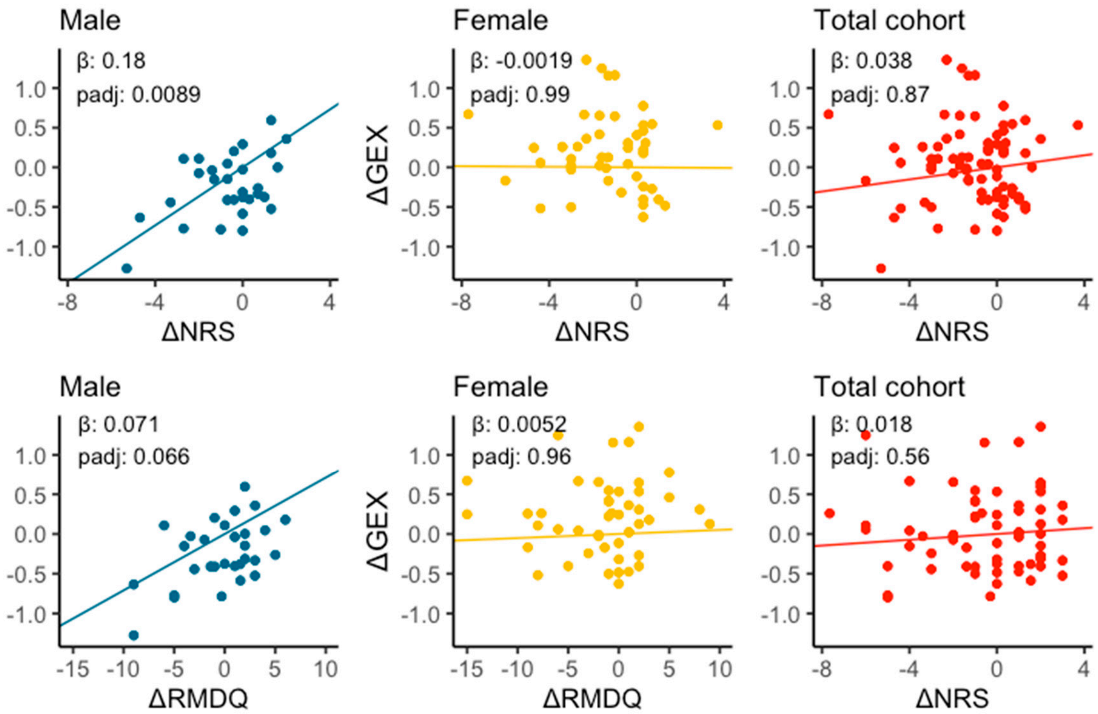

Supplementary Figure S6: Change in expression of IL-6 correlates to ΔNRS in males, but not in females or in mixed-sex cohorts, or to ΔRMDQ in any cohort.

COMT

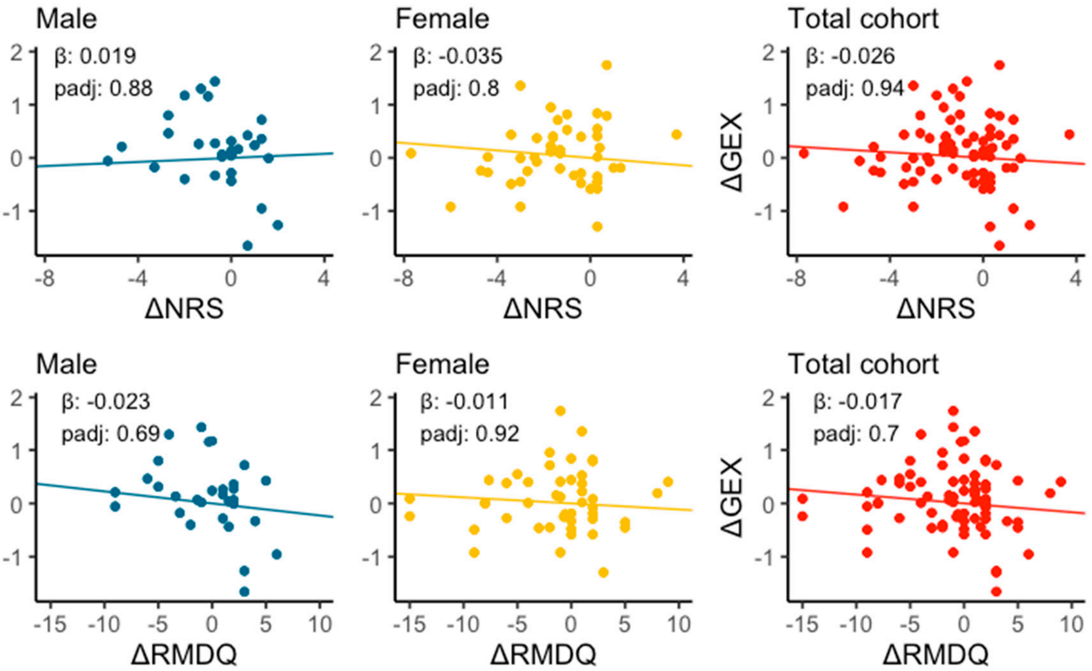

Supplementary Figure S7: No significant correlation between ΔNRS and ΔRMDQ and change in expression for COMT in males, females or mixed-sex cohorts.
