# Supplementary material for: Gene Expression Correlates with Disability and Pain Intensity in Patients with Chronic Low Back Pain and Modic Changes in a Sex-Specific Manner
Source: Int J Mol Sci. 2025 Jan 18;26(2):800. doi: 10.3390/ijms26020800 (PMC11766089; doi:10.3390/ijms26020800)
Supplement: Supplementary file 1 [file ijms-26-00800-s001.zip › Supplementary_table_s1.pdf]

Supplementary Table S1: Number of patients with non-missing data for A) RMDQ and NRS and B) ΔRMDQ and ΔNRS

A)

	Time point	RMDQ	NRS
**Females**	Day 0	26	25
	Day 100	24	24
	1y	25	25
**Males**	Day 0	15	15
	Day 100	15	15
	1y	15	15

B)

	Time interval	ΔRMDQ	ΔNRS
**Females**	Day 0 – day 100	23	23
	Day 0 – 1y	24	24
**Males**	Day 0 – day 100	15	15
	Day 0 – 1y	15	15
